# Pharyngeal Lavage Lymphocytosis in Patients with Obstructive Sleep Apnea: A Preliminary Observation

**DOI:** 10.1371/journal.pone.0016277

**Published:** 2011-01-19

**Authors:** Hans-Peter Hauber, Stefan Rüller, Ernst Müller, Eike Hansen, Peter Zabel

**Affiliations:** Sleep Disorder Unit, Medical Clinic, Research Center Borstel, Borstel, Germany; University of Pittsburgh, United States of America

## Abstract

**Background:**

Upper airway inflammation has been previously demonstrated in obstructive sleep apnea (OSA). However, investigation has been hampered by the necessity of invasive tissue biopsies.

**Objective:**

To evaluate the pharyngeal lavage (PHAL) as a new tool to analyze mucosal inflammation in the pharynx of patients with sleep-related disordered breathing.

**Patients and Methods:**

36 patients with a diagnosis of OSA, 14 patients with heavy snorer syndrome (HS) or body position dependent OSA (bd-OSA), and 14 healthy volunteers underwent PHAL. Inflammatory cell counts were compared.

**Results:**

Neutrophils were the predominant cells in PHAL in all groups (94.3%±0.7%, 98.5%±0.6%, 94.3%±0.7%, and 96.2%±1.4%). OSA patients had significantly increased numbers of lymphocytes (3.2%±0.4%) compared to bd-OSA/HS and controls group (0.5%±0.1% and 0.6%±0.2%, respectively; P<0.05). Patients with moderate to severe OSA had significantly higher numbers of lymphocytes compared to patients with mild OSA (P<0.05).

**Conclusions:**

Data from this study suggest that PHAL is a feasible tool to investigate upper airway inflammation in OSA. In addition, PHAL demonstrates lymphocytic inflammation of the pharynx in OSA patients. Future studies are warranted to evaluate whether PHAL can be used to monitor disease and whether lymphocytic inflammation is affected by OSA treatment.

## Introduction

Obstructive sleep apnea (OSA) is a common disorder in the Western World affecting 4% of all men and 2% of all women [1]. Recurrent collapse of the upper airways during sleep causes recurrent hypopnea and apnea resulting in hypoxaemia, arrhythmia and rise of blood pressure as well as arousal. OSA has been shown to be associated with cardiac and vascular diseases [2]–[4]. Endothelial dysfunction and systemic inflammation are considered as important underlying pathophysiologic conditions [5], [6].

In addition to systemic inflammation previous studies including work from our own laboratory have demonstrated local inflammation in the upper airway mucosa of patients with OSA [7]–[9]. Leukocyte numbers are increased in the mucosa of both pharynx and nose [7], [8]. It is unclear whether this inflammation is due to an intrinsic factor in the pathogenesis of OSA or due to mechanical stress caused by repetitive collapse of the airways. The present hypothesis favours stress-induced inflammation [10]. Recent experiments with an animal model of recurrent collapse of the airways further support this hypothesis [11].

Until recently investigations of local inflammatory processes in the upper airway mucosa in OSA patients were hampered by the need to perform invasive tissue biopsies. These biopsies are uncomfortable for the patient especially when taken at different time points. Moreover, biopsies may also cause serious side effects (eg. bleeding, infection). We recently reported a new method for non-invasive examination of inflammatory cells in the pharynx, the so called pharyngeal lavage (PHAL) [9].

The intermediate collapsibility of the nonapneic's upper airways relative to healthy controls and OSA patients has led to the concept that snoring and OSA are part of a spectrum of sleep-related breathing disorders. About 10% of all snorers have OSA [12].

Since there is a difference in leukocyte numbers between healthy controls and OSA patients [7], [9] we wondered whether lymphocyte counts would be elevated in snoring patients or patients with body position-dependent OSA (bd-OSA). We hypothesized that local inflammation may be also present but show lower intensity snorers and patients with bd-OSA. We speculated that local inflammation may develop with increasing severity of sleep-related disordered breathing and upper airway collapse. To test this hypothesis PHAL was performed to analyze local inflammation of the upper airways in patients with OSA, bd-OSA/snoring patients and in healthy controls.

## Materials and Methods

### Patients and controls

36 patients with a diagnosis of OSA, 14 patients with heavy snorer syndrome or body position dependent OSA (bd-OSA), and 14 healthy volunteers were included into the study. Ethics approval was obtained by the ethics committee of the university of Lübeck. Verbal informed consent was obtained from all patients and volunteers. This was documented in written form in accordance with ethical guidelines of the local ethics committee. Part of the study population was already used for analysis in another publication (14 patients with OSA and 11 controls, 9). Table 1 summarizes the demographics of the patient groups and the controls. Healthy controls did not undergo standard polysomnography (PSG) and none had reported sleep-related breathing disorders. Patients with severe concomitant disease (eg. heart failure, liver failure, kidney failure), acute or chronic inflammatory disease of the airways or use of drugs that can affect sleep (eg. sedatives, anxiolytics, illegal drugs) were excluded from the study. Controls and patients were non-smokers. Severity of OSA was classified according to the RDI (respiratory distress index): RDI 5-10/h mild, RDI 11-15/h moderate, RDI >15/h severe. Patients were classified as having body position-dependent OSA if the RDI was >5/h in supine position and <5/h in any other body position. Patients were classified as heavy snorers (HS) if the snoring index was >20/h and if obstructive hyponeas/apneas were present only in supine position or RDI <5/h [13], [14].

**Table 1:** Demographic characteristics of patient groups and controls.

	OSA	bd-OSA/HS	Control
Number (male/female)	36 (26/10)	14 (12/2)	14 (5/9)
Mean age (years)	52.1±2.1	49.4±2.2	31.8±4.7
Mean RDI	33.4±3.6	19.3±3.3*	n. d.
Mean ODI	28.8±3.8	15.9±3.5*	n. d.
Mean ESS-Score	18.5±1.4	12.3±1.9*	n. d.

OSA: Obstructive sleep apnea. Bd-OSA: body-dependent OSA. HS: heavy snorer. RDI: respiratory distress index. ODI: oxygen desaturation index. ESS: Epworth sleepiness scale.

*: P<0.05 vs OSA.

### Pharyngeal lavage

Pharnygeal lavage (PHAL) was performed in the morning after waking up. Patients and controls rinsed their mouth with sterile saline at room temperature. After that they gargled with 50 ml of sterile saline in several fractions for 5 to 10 sec each. Patients and controls spit the saline in to a sterile container for sample collection. Samples underwent centrifugation with 1500 RPM for 10 min. The cell pellet was resuspended into sterile saline and cytospins were made (centrifugation for 750 RPM for 5 min). Cytospins were stained with Hemacolor fast stain (Merck, Darmstadt, Germany) for further analysis.

### Cell count and differential cell count

Squamous cells that represented the largest quantity of cells on the cytospins were not further included into analysis. 200 to 400 inflammatory cells were counted per cytospins for differential cell counts. Counting was performed by two experienced investigators without blinded to clinical data. The within-observer coefficient of variation for repeated measures was less than 5%.

### Statistical analysis

The difference in the number of inflammatory cells between the groups were compared with the nonparametric Kruskal-Wallis test. Statistically significant differences between groups were subsequently analyzed with the Mann-Whitney *U* test (Systat version 7.0; SPSS, Chicago, Ill). Results are expressed as means±SEM and are significant at a *P* value of less than .05.

## Results

### Characterization of patients and controls

Table 1 summarizes the demographics in the different groups. There was a significant difference in the RDI (respiratory distress index), in the ODI (oxygen desaturation index) and in the Epworth sleepiness scale score between OSA patients and bd-OSA patients/HS (P<0.05).

### Inflammatory cell counts in the pharyngeal lavage

**Figure 1:**
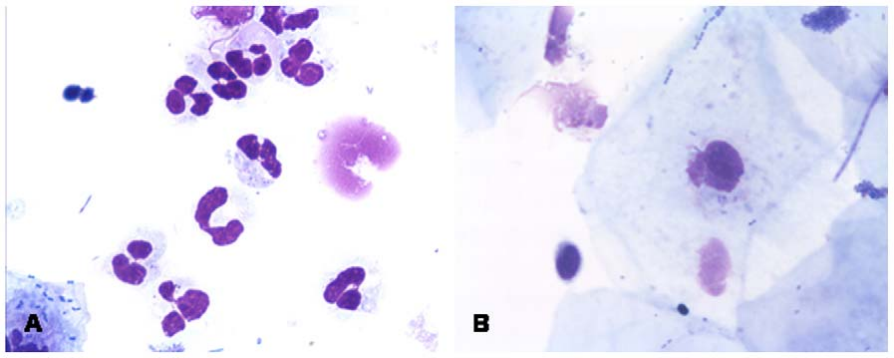
Microscopy of pharyngeal lavage showing neutrophils (A) and squamous cells and lymphocytes (B). Hemacolor fats stain. Original magnification x400.

**Figure 2:**
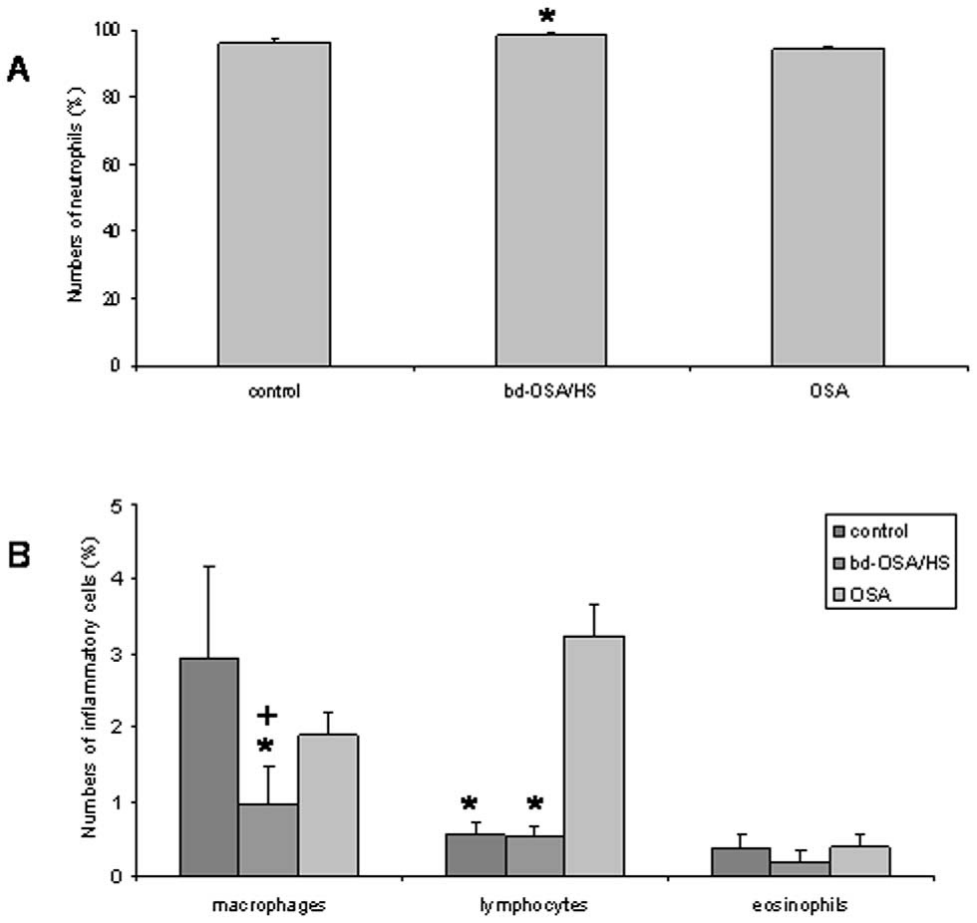
Numbers of neutrophils (A) and other inflammatory cells (B) in the pharhygeal lavage differential cell counts from controls and patient groups. Bars represent mean values+SEM. OSA: obstructive sleep apnea. Bd-OSA: body position dependent-OSA. HS: heavy snorer. *: P<0.05 vs OSA. +: P<0.05 vs control.

As expected squamous cells represented the majority of cells in the PHAL. They were not further analyzed because they were most probably shed from the airways while performing the PHAL. Neutrophils were the most prominent inflammatory cells in all three groups. Figure 1 shows original microscopic images of PHAL. The numbers of neutrophils were significantly higher in the bd-OSA/HS group compared to the OSA group (98.5%±0.6% vs 94.3%±0.7%; P<0.05). There was no significant difference compared to the control group (96.2%±1.4%; P>0.05) (Fig. 2A). The numbers of lymphocytes were significantly increased in the OSA group (3.2%±0.4%) compared to the bd-OSA/HS and compared to the control group (0.5%±0.1% and 0.6%±0.2%, respectively) (P<0.05) (Fig. 2B). The percentage of macrophages was significantly lower in the bd-OSA/HS (0.9%±0.5%) compared to the control group and to the OSA group (2.9%±1.2% and 1.9%±0.3%, respectively) (P<0.05). There was no significant difference in the numbers of eosinophils between the three groups (control: 0.4%±0.2%, bd-OSA/HS: 0.2%±0.2%, OSA: 0.4%±0.2%) (P>0.05).

### Lymphocytes and severity of sleep-related breathing disorders

**Figure 3:**
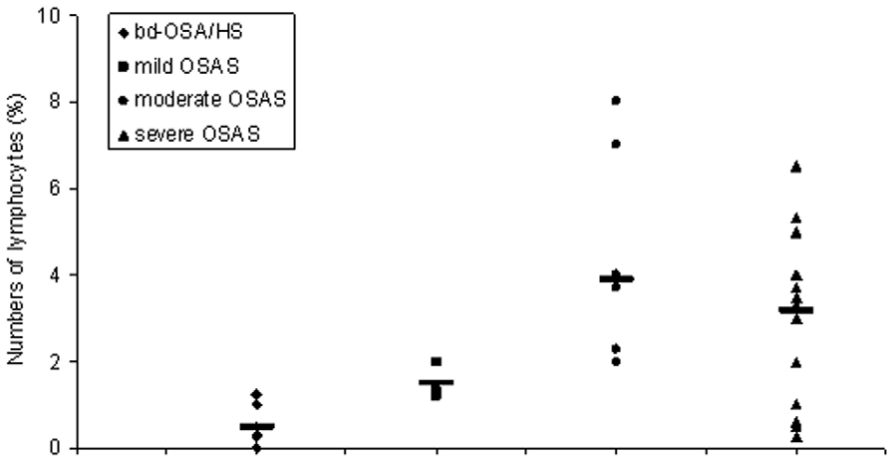
Numbers of lymphocytes according to severity of OSA. Dots represent individual values. Lines represent mean values. *: P<0.05 vs mild OSA. +: P<0.05 vs mild, moderate and sever OSA.

The numbers of lymphocytes were significantly increased in the OSA patients with moderate and severe disease (3.9%±0.8% and 3.2%±0.6%, respectively) compared to patients with mild disease (1.5%±0.3%) (P<0.05). There was no statistically significant difference between patients with moderate and severe disease (P>0.05). The percentage of lymphocytes was significantly higher in patients with OSA than in patients with bd-OSA or HS regardless of the severity of OSA (P<0.05) (Fig. 3). However, no significant correlation between lymphocytes and the severity of OSA was observed (P>0.05).

## Discussion

In the present study PHAL was used to evaluate local inflammation in the upper airways of patients with OSA, bd-OSA patients/HS and healthy controls. We found significantly increased numbers of lymphocytes in patients with OSA compared to both patients with bd-OSA/HS and healthy controls.

Information on airway inflammation and inflammatory cell in the airway mucosa in patients with sleep apnea is sparse [7], [15]. Studies by Sekosan and co-workers and by Boyd and colleagues demonstrated leukocyte infiltration and lymphocytic inflammation in the mucosa of patients with OSA [7], [15]. In our study we also found increased numbers of lymphocytes in OSA patients. While the above mentioned studies only compared OSA and healthy controls we also analyzed the PHAL of patients with bd-OSA and HS. Interestingly, there was no significant difference in the numbers of PHAL lymphocytes between normal controls and patients with bd-OSA/HS. Thus bd-OSA and heavy snoring may be a different entity from OSA or they may represent the first early stages of a disease process leading eventually to OSA. To our knowledge our study is the first to investigate and compare upper airway inflammation by PHAL in these three groups.

At present it is not clear whether local inflammation in OSA is the cause of pharyngeal collapse or whether it results from this. A recent study in an animal model of cycling airway collapse supports the hypothesis that mechanic traumata due to airway collapse lead to mucosal inflammation [11]. If snoring is considered as a first step in the development of OSA our data agree with those studies and imply that development of lymphocytic mucosal inflammation may be a crucial factor. Another possibility is that snoring and OSA represent different ends of a spectrum of sleep-related breathing disorders. Long term follow-up studies of snorers may help to clarify this point.

A disadvantage of PHAL is that it cannot be easily standardized. Moreover, we only used percentages of inflammatory cells and ignored squamous cells. However, in bronchoalveolar lavage a widely used and accepted technique the differential cell counts and the percentage of inflammatory cells provides the most important information on inflammation.

One major point of weakness of the study is that no tissue biopsies were obtained for histologic examination and verification of tissue inflammation. Tissue biopsies are potentially harmful for the patient. Our aim was to investigate whether a non-invasive method could detect local inflammation in the upper airways in patients with sleep-related disordered breathing. Previous experiments in our own lab showed that differential cytology in PHAL correlated well with inflammatory cell profiles in mucosal biopsies (unpublished observation). Taking into account previous data from the literature that demonstrated increased numbers of leucocytes in the airway mucosa of patients with OSA [7] we think that PHAL indeed reflects local airway inflammation.

In conclusion the present study describes PHAL as a new tool to investigate mucosal inflammation of the upper airways in patients with obstructive sleep apnea. In addition, the present data demonstrate that lymphocytic inflammation is present in the pharyngeal muocsa in OSA but not in bd-OSA/HS. Future studies are warranted to evaluate whether PHAL can be used to monitor disease and whether lymphocytic inflammation of the upper airways is affected by treatment with continous positive airway pressure (CPAP).
